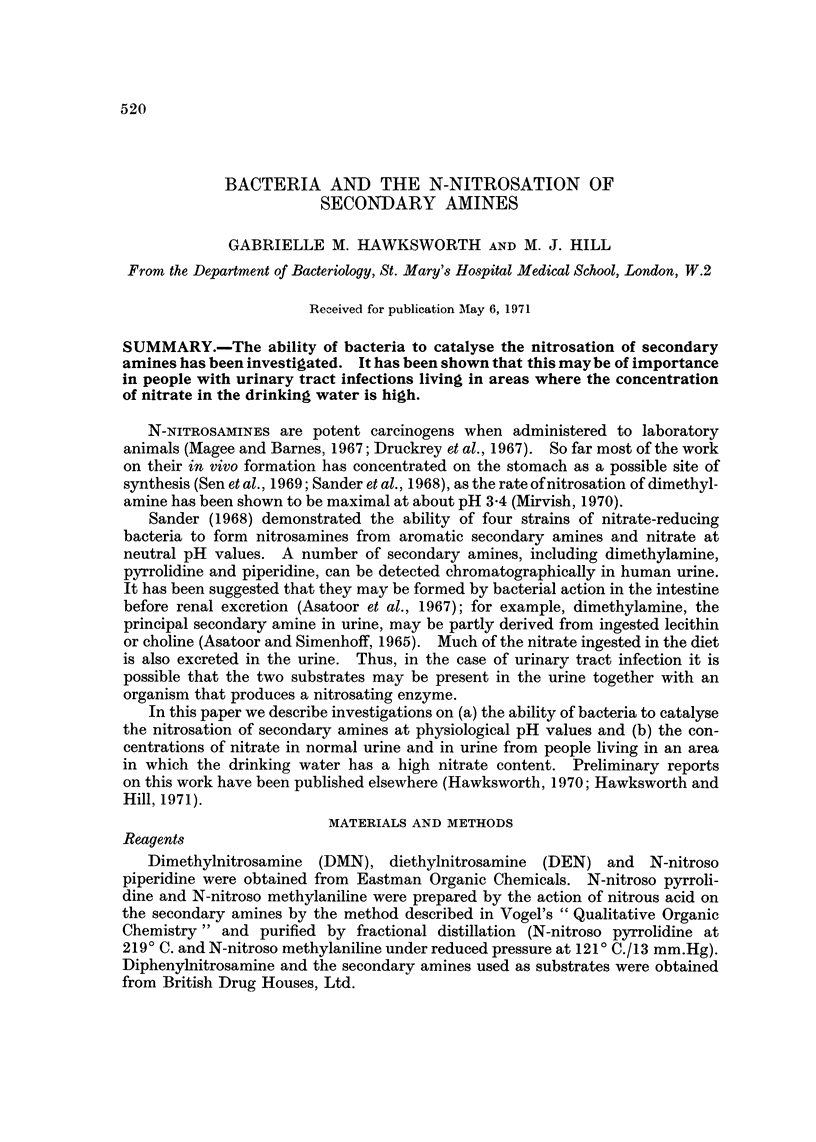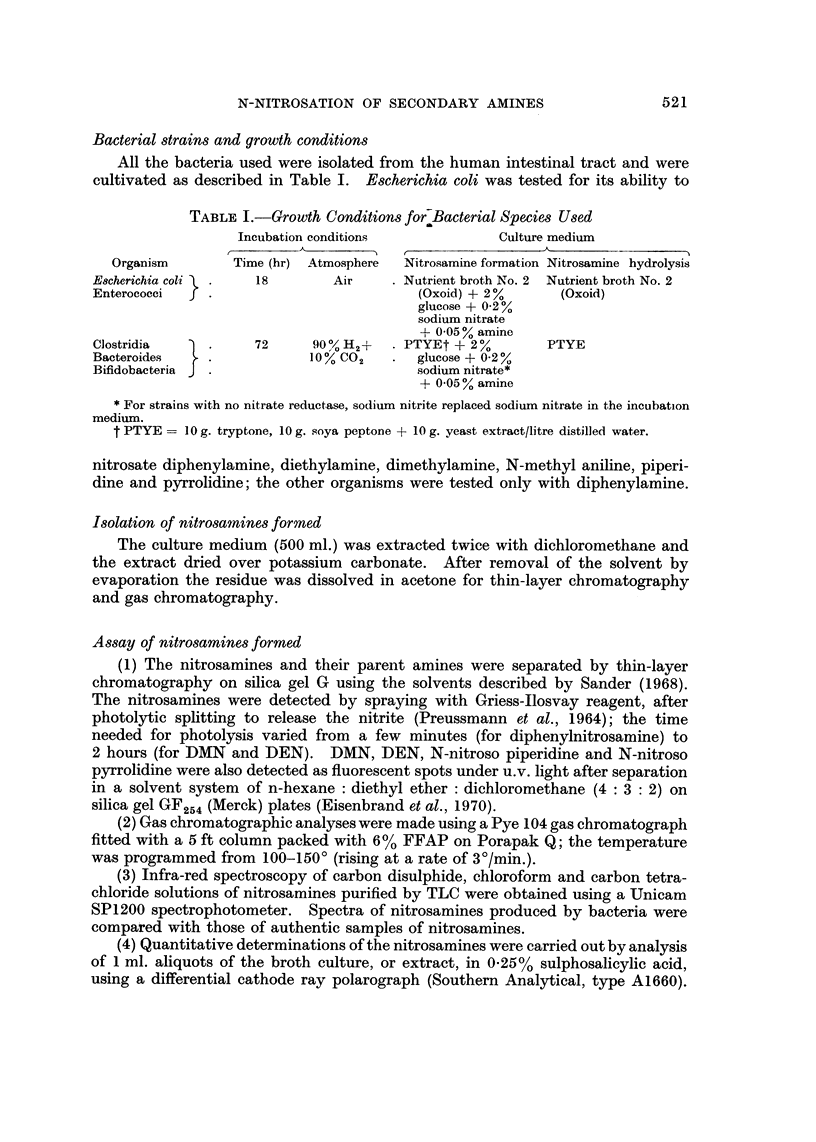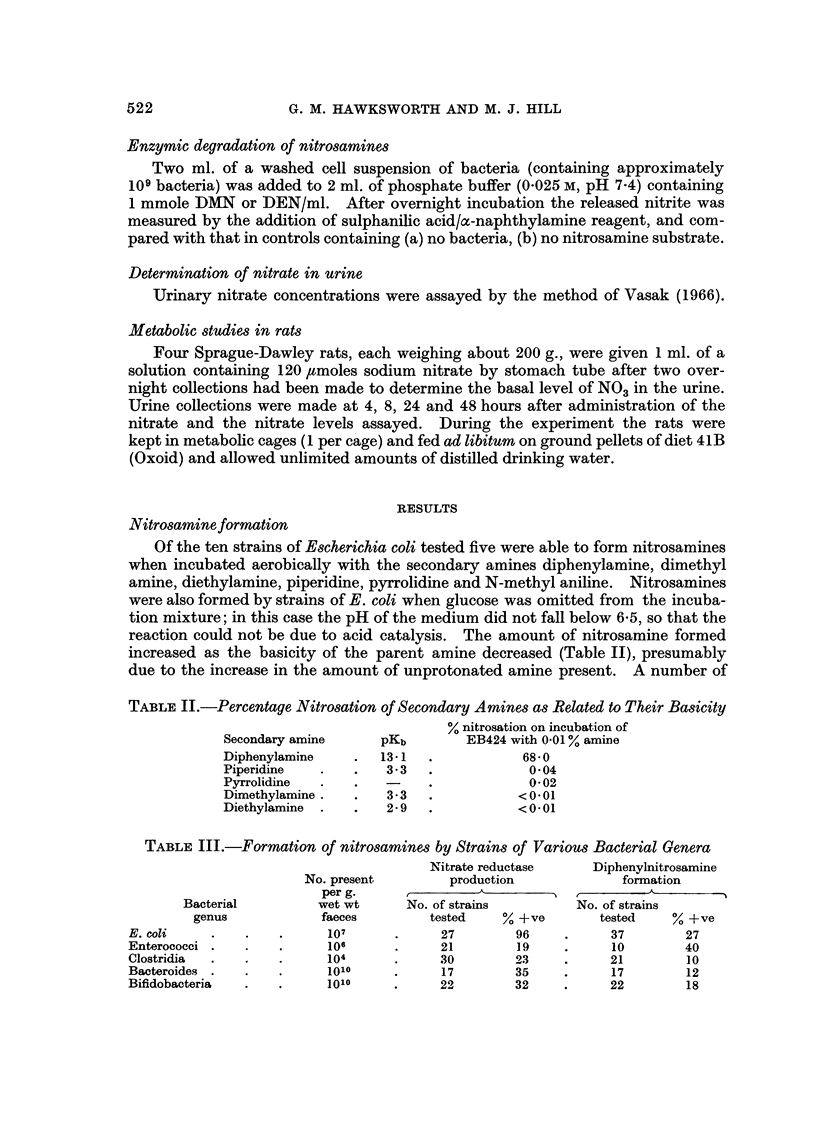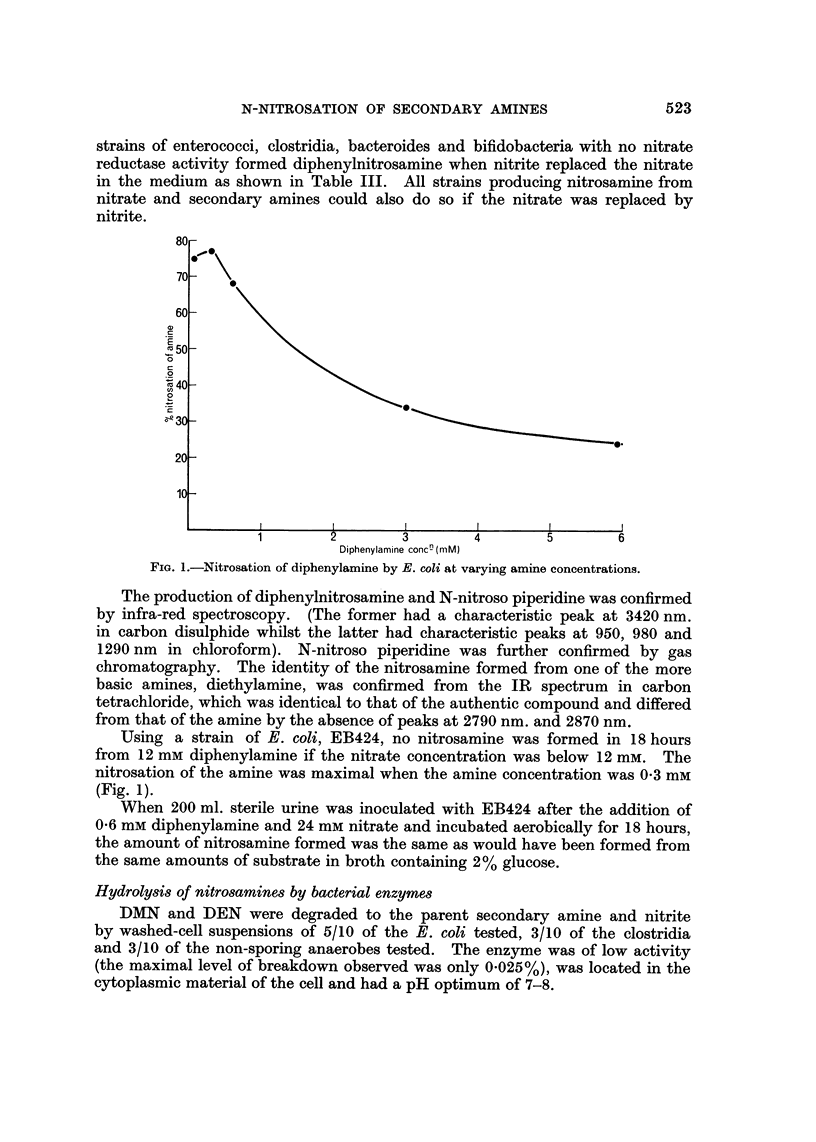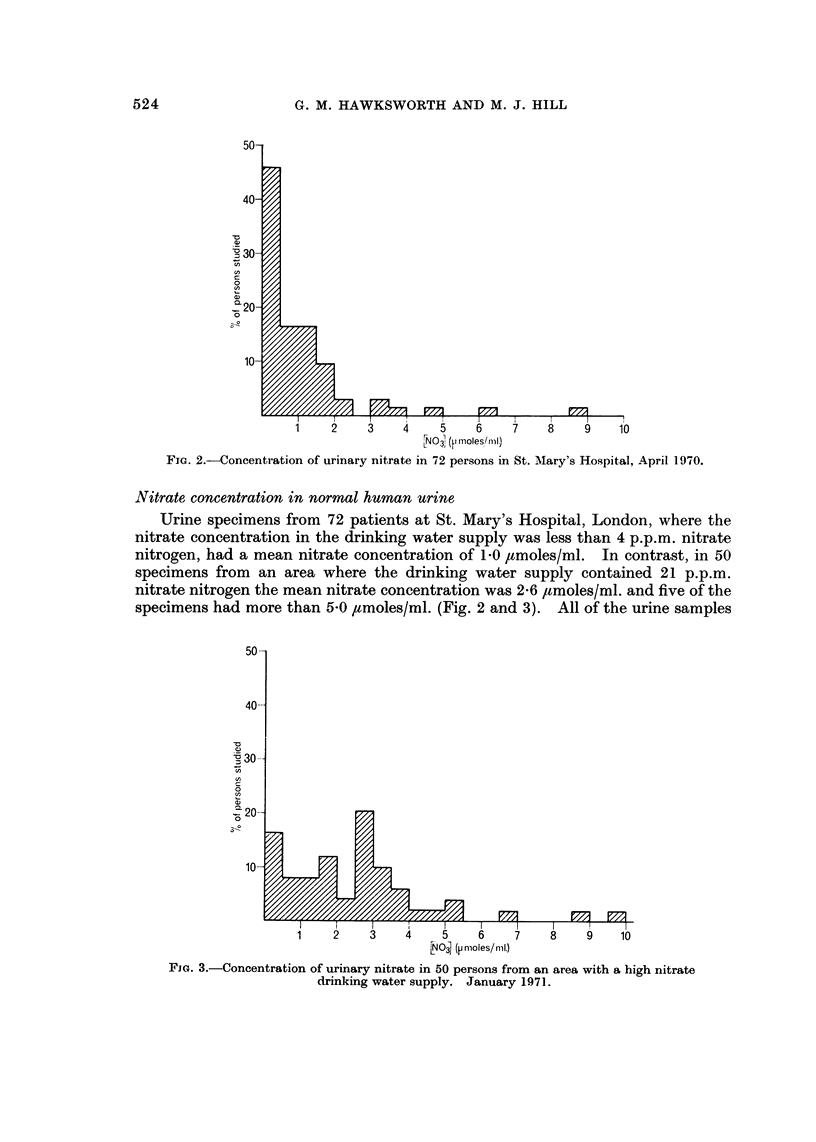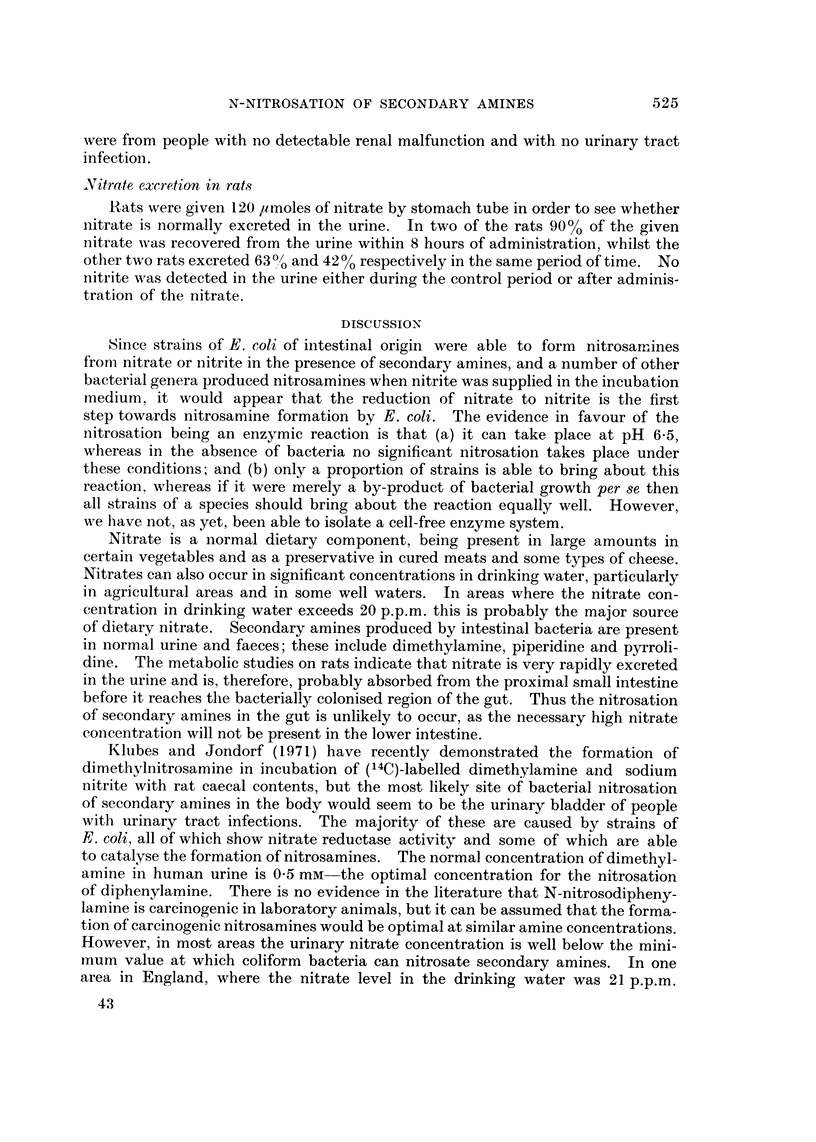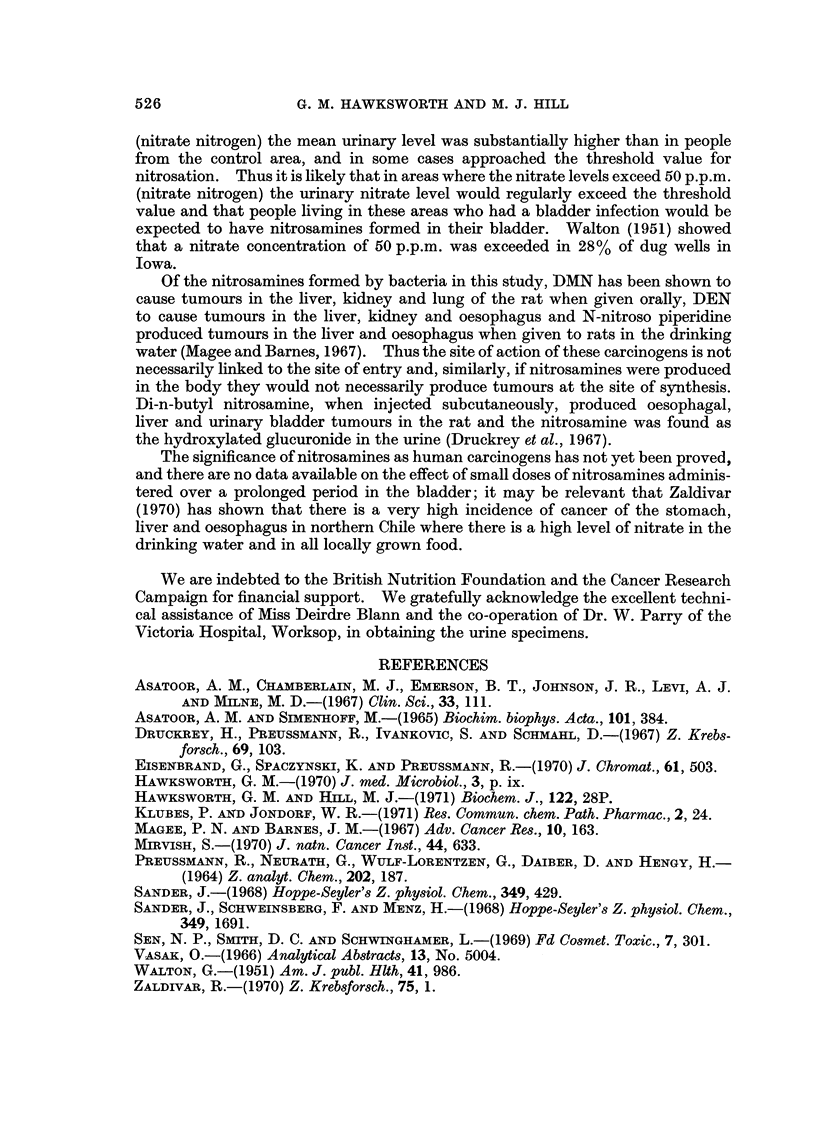# Bacteria and the N-Nitrosation of Secondary Amines

**DOI:** 10.1038/bjc.1971.66

**Published:** 1971-09

**Authors:** Gabrielle M. Hawksworth, M. J. Hill

## Abstract

The ability of bacteria to catalyse the nitrosation of secondary amines has been investigated. It has been shown that this may be of importance in people with urinary tract infections living in areas where the concentration of nitrate in the drinking water is high.


### BACTERIA AND THE N-NITROSATION OF SECONDARY AMINES

GABRIELLE M. HAWKSWORTH AND M. J. HILL

*From the Department of Bacteriology, St. Mary's Hospital Medical School, London, W.2*

Received for publication May 6, 1971

**SUMMARY.—The ability of bacteria to catalyse the nitrosation of secondary amines has been investigated. It has been shown that this may be of importance in people with urinary tract infections living in areas where the concentration of nitrate in the drinking water is high.**

N-NITROSAMINES are potent carcinogens when administered to laboratory animals (Magee and Barnes, 1967; Druckrey *et al.*, 1967). So far most of the work on their *in vivo* formation has concentrated on the stomach as a possible site of synthesis (Sen *et al.*, 1969; Sander *et al.*, 1968), as the rate of nitrosation of dimethylamine has been shown to be maximal at about pH 3·4 (Mirvish, 1970).

Sander (1968) demonstrated the ability of four strains of nitrate-reducing bacteria to form nitrosamines from aromatic secondary amines and nitrate at neutral pH values. A number of secondary amines, including dimethylamine, pyrrolidine and piperidine, can be detected chromatographically in human urine. It has been suggested that they may be formed by bacterial action in the intestine before renal excretion (Asatoor *et al.*, 1967); for example, dimethylamine, the principal secondary amine in urine, may be partly derived from ingested lecithin or choline (Asatoor and Simenhoff, 1965). Much of the nitrate ingested in the diet is also excreted in the urine. Thus, in the case of urinary tract infection it is possible that the two substrates may be present in the urine together with an organism that produces a nitrosating enzyme.

In this paper we describe investigations on (a) the ability of bacteria to catalyse the nitrosation of secondary amines at physiological pH values and (b) the concentrations of nitrate in normal urine and in urine from people living in an area in which the drinking water has a high nitrate content. Preliminary reports on this work have been published elsewhere (Hawksworth, 1970; Hawksworth and Hill, 1971).

### MATERIALS AND METHODS

### *Reagents*

Dimethylnitrosamine (DMN), diethylnitrosamine (DEN) and N-nitroso piperidine were obtained from Eastman Organic Chemicals. N-nitroso pyrrolidine and N-nitroso methylaniline were prepared by the action of nitrous acid on the secondary amines by the method described in Vogel's " Qualitative Organic Chemistry " and purified by fractional distillation (N-nitroso pyrrolidine at 219° C. and N-nitroso methylaniline under reduced pressure at 121° C./13 mm.Hg). Diphenylnitrosamine and the secondary amines used as substrates were obtained from British Drug Houses, Ltd.

*Bacterial strains and growth conditions*

All the bacteria used were isolated from the human intestinal tract and were cultivated as described in Table I. *Escherichia coli* was tested for its ability to nitrosate diphenylamine, diethylamine, dimethylamine, N-methyl aniline, piperidine and pyrrolidine; the other organisms were tested only with diphenylamine.

TABLE I.—*Growth Conditions for Bacterial Species Used*

| | Incubation conditions | | Culture medium | |
|---|---|---|---|---|
| Organism | Time (hr) | Atmosphere | Nitrosamine formation | Nitrosamine hydrolysis |
| *Escherichia coli*, Enterococci | 18 | Air | Nutrient broth No. 2 (Oxoid) + 2% glucose + 0·2% sodium nitrate + 0·05% amine | Nutrient broth No. 2 (Oxoid) |
| Clostridia, Bacteroides, Bifidobacteria | 72 | 90% $H_2$+ 10% $CO_2$ | PTYE† + 2% glucose + 0·2% sodium nitrate* + 0·05% amine | PTYE |

* For strains with no nitrate reductase, sodium nitrite replaced sodium nitrate in the incubation medium.

† PTYE = 10 g. tryptone, 10 g. soya peptone + 10 g. yeast extract/litre distilled water.

*Isolation of nitrosamines formed*

The culture medium (500 ml.) was extracted twice with dichloromethane and the extract dried over potassium carbonate. After removal of the solvent by evaporation the residue was dissolved in acetone for thin-layer chromatography and gas chromatography.

*Assay of nitrosamines formed*

(1) The nitrosamines and their parent amines were separated by thin-layer chromatography on silica gel G using the solvents described by Sander (1968). The nitrosamines were detected by spraying with Griess-Ilosvay reagent, after photolytic splitting to release the nitrite (Preussmann *et al.*, 1964); the time needed for photolysis varied from a few minutes (for diphenylnitrosamine) to 2 hours (for DMN and DEN). DMN, DEN, N-nitroso piperidine and N-nitroso pyrrolidine were also detected as fluorescent spots under u.v. light after separation in a solvent system of n-hexane : diethyl ether : dichloromethane (4 : 3 : 2) on silica gel $GF_{254}$ (Merck) plates (Eisenbrand *et al.*, 1970).

(2) Gas chromatographic analyses were made using a Pye 104 gas chromatograph fitted with a 5 ft column packed with 6% FFAP on Porapak Q; the temperature was programmed from 100–150° (rising at a rate of 3°/min.).

(3) Infra-red spectroscopy of carbon disulphide, chloroform and carbon tetrachloride solutions of nitrosamines purified by TLC were obtained using a Unicam SP1200 spectrophotometer. Spectra of nitrosamines produced by bacteria were compared with those of authentic samples of nitrosamines.

(4) Quantitative determinations of the nitrosamines were carried out by analysis of 1 ml. aliquots of the broth culture, or extract, in 0·25% sulphosalicylic acid, using a differential cathode ray polarograph (Southern Analytical, type A1660).

*Enzymic degradation of nitrosamines*

Two ml. of a washed cell suspension of bacteria (containing approximately $10^9$ bacteria) was added to 2 ml. of phosphate buffer (0·025 M, pH 7·4) containing 1 mmole DMN or DEN/ml. After overnight incubation the released nitrite was measured by the addition of sulphanilic acid/$\alpha$-naphthylamine reagent, and compared with that in controls containing (a) no bacteria, (b) no nitrosamine substrate.

*Determination of nitrate in urine*

Urinary nitrate concentrations were assayed by the method of Vasak (1966).

*Metabolic studies in rats*

Four Sprague-Dawley rats, each weighing about 200 g., were given 1 ml. of a solution containing 120 $\mu$moles sodium nitrate by stomach tube after two overnight collections had been made to determine the basal level of $NO_3$ in the urine. Urine collections were made at 4, 8, 24 and 48 hours after administration of the nitrate and the nitrate levels assayed. During the experiment the rats were kept in metabolic cages (1 per cage) and fed *ad libitum* on ground pellets of diet 41B (Oxoid) and allowed unlimited amounts of distilled drinking water.

### RESULTS

*Nitrosamine formation*

Of the ten strains of *Escherichia coli* tested five were able to form nitrosamines when incubated aerobically with the secondary amines diphenylamine, dimethyl amine, diethylamine, piperidine, pyrrolidine and N-methyl aniline. Nitrosamines were also formed by strains of *E. coli* when glucose was omitted from the incubation mixture; in this case the pH of the medium did not fall below 6·5, so that the reaction could not be due to acid catalysis. The amount of nitrosamine formed increased as the basicity of the parent amine decreased (Table II), presumably due to the increase in the amount of unprotonated amine present. A number of

TABLE II.—*Percentage Nitrosation of Secondary Amines as Related to Their Basicity*

| Secondary amine | $pK_b$ | % nitrosation on incubation of EB424 with 0·01% amine |
|---|---|---|
| Diphenylamine | 13·1 | 68·0 |
| Piperidine | 3·3 | 0·04 |
| Pyrrolidine | — | 0·02 |
| Dimethylamine | 3·3 | <0·01 |
| Diethylamine | 2·9 | <0·01 |

TABLE III.—*Formation of nitrosamines by Strains of Various Bacterial Genera*

| Bacterial genus | No. present per g. wet wt faeces | Nitrate reductase production: No. of strains tested | Nitrate reductase production: % +ve | Diphenylnitrosamine formation: No. of strains tested | Diphenylnitrosamine formation: % +ve |
|---|---|---|---|---|---|
| *E. coli* | $10^7$ | 27 | 96 | 37 | 27 |
| Enterococci | $10^6$ | 21 | 19 | 10 | 40 |
| Clostridia | $10^4$ | 30 | 23 | 21 | 10 |
| Bacteroides | $10^{10}$ | 17 | 35 | 17 | 12 |
| Bifidobacteria | $10^{10}$ | 22 | 32 | 22 | 18 |

strains of enterococci, clostridia, bacteroides and bifidobacteria with no nitrate reductase activity formed diphenylnitrosamine when nitrite replaced the nitrate in the medium as shown in Table III. All strains producing nitrosamine from nitrate and secondary amines could also do so if the nitrate was replaced by nitrite.

FIG. 1.—Nitrosation of diphenylamine by *E. coli* at varying amine concentrations.

The production of diphenylnitrosamine and N-nitroso piperidine was confirmed by infra-red spectroscopy. (The former had a characteristic peak at 3420 nm. in carbon disulphide whilst the latter had characteristic peaks at 950, 980 and 1290 nm in chloroform). N-nitroso piperidine was further confirmed by gas chromatography. The identity of the nitrosamine formed from one of the more basic amines, diethylamine, was confirmed from the IR spectrum in carbon tetrachloride, which was identical to that of the authentic compound and differed from that of the amine by the absence of peaks at 2790 nm. and 2870 nm.

Using a strain of *E. coli*, EB424, no nitrosamine was formed in 18 hours from 12 mM diphenylamine if the nitrate concentration was below 12 mM. The nitrosation of the amine was maximal when the amine concentration was 0·3 mM (Fig. 1).

When 200 ml. sterile urine was inoculated with EB424 after the addition of 0·6 mM diphenylamine and 24 mM nitrate and incubated aerobically for 18 hours, the amount of nitrosamine formed was the same as would have been formed from the same amounts of substrate in broth containing 2% glucose.

*Hydrolysis of nitrosamines by bacterial enzymes*

DMN and DEN were degraded to the parent secondary amine and nitrite by washed-cell suspensions of 5/10 of the *E. coli* tested, 3/10 of the clostridia and 3/10 of the non-sporing anaerobes tested. The enzyme was of low activity (the maximal level of breakdown observed was only 0·025%), was located in the cytoplasmic material of the cell and had a pH optimum of 7–8.

FIG. 2.—Concentration of urinary nitrate in 72 persons in St. Mary's Hospital, April 1970.

*Nitrate concentration in normal human urine*

Urine specimens from 72 patients at St. Mary's Hospital, London, where the nitrate concentration in the drinking water supply was less than 4 p.p.m. nitrate nitrogen, had a mean nitrate concentration of 1·0 μmoles/ml. In contrast, in 50 specimens from an area where the drinking water supply contained 21 p.p.m. nitrate nitrogen the mean nitrate concentration was 2·6 μmoles/ml. and five of the specimens had more than 5·0 μmoles/ml. (Fig. 2 and 3). All of the urine samples

FIG. 3.—Concentration of urinary nitrate in 50 persons from an area with a high nitrate drinking water supply. January 1971.

were from people with no detectable renal malfunction and with no urinary tract infection.

*Nitrate excretion in rats*

Rats were given 120 $\mu$moles of nitrate by stomach tube in order to see whether nitrate is normally excreted in the urine. In two of the rats 90% of the given nitrate was recovered from the urine within 8 hours of administration, whilst the other two rats excreted 63% and 42% respectively in the same period of time. No nitrite was detected in the urine either during the control period or after administration of the nitrate.

### DISCUSSION

Since strains of *E. coli* of intestinal origin were able to form nitrosamines from nitrate or nitrite in the presence of secondary amines, and a number of other bacterial genera produced nitrosamines when nitrite was supplied in the incubation medium, it would appear that the reduction of nitrate to nitrite is the first step towards nitrosamine formation by *E. coli*. The evidence in favour of the nitrosation being an enzymic reaction is that (a) it can take place at pH 6·5, whereas in the absence of bacteria no significant nitrosation takes place under these conditions; and (b) only a proportion of strains is able to bring about this reaction, whereas if it were merely a by-product of bacterial growth *per se* then all strains of a species should bring about the reaction equally well. However, we have not, as yet, been able to isolate a cell-free enzyme system.

Nitrate is a normal dietary component, being present in large amounts in certain vegetables and as a preservative in cured meats and some types of cheese. Nitrates can also occur in significant concentrations in drinking water, particularly in agricultural areas and in some well waters. In areas where the nitrate concentration in drinking water exceeds 20 p.p.m. this is probably the major source of dietary nitrate. Secondary amines produced by intestinal bacteria are present in normal urine and faeces; these include dimethylamine, piperidine and pyrrolidine. The metabolic studies on rats indicate that nitrate is very rapidly excreted in the urine and is, therefore, probably absorbed from the proximal small intestine before it reaches the bacterially colonised region of the gut. Thus the nitrosation of secondary amines in the gut is unlikely to occur, as the necessary high nitrate concentration will not be present in the lower intestine.

Klubes and Jondorf (1971) have recently demonstrated the formation of dimethylnitrosamine in incubation of ($^{14}$C)-labelled dimethylamine and sodium nitrite with rat caecal contents, but the most likely site of bacterial nitrosation of secondary amines in the body would seem to be the urinary bladder of people with urinary tract infections. The majority of these are caused by strains of *E. coli*, all of which show nitrate reductase activity and some of which are able to catalyse the formation of nitrosamines. The normal concentration of dimethylamine in human urine is 0·5 mM—the optimal concentration for the nitrosation of diphenylamine. There is no evidence in the literature that N-nitrosodiphenylamine is carcinogenic in laboratory animals, but it can be assumed that the formation of carcinogenic nitrosamines would be optimal at similar amine concentrations. However, in most areas the urinary nitrate concentration is well below the minimum value at which coliform bacteria can nitrosate secondary amines. In one area in England, where the nitrate level in the drinking water was 21 p.p.m.

(nitrate nitrogen) the mean urinary level was substantially higher than in people from the control area, and in some cases approached the threshold value for nitrosation. Thus it is likely that in areas where the nitrate levels exceed 50 p.p.m. (nitrate nitrogen) the urinary nitrate level would regularly exceed the threshold value and that people living in these areas who had a bladder infection would be expected to have nitrosamines formed in their bladder. Walton (1951) showed that a nitrate concentration of 50 p.p.m. was exceeded in 28% of dug wells in Iowa.

Of the nitrosamines formed by bacteria in this study, DMN has been shown to cause tumours in the liver, kidney and lung of the rat when given orally, DEN to cause tumours in the liver, kidney and oesophagus and N-nitroso piperidine produced tumours in the liver and oesophagus when given to rats in the drinking water (Magee and Barnes, 1967). Thus the site of action of these carcinogens is not necessarily linked to the site of entry and, similarly, if nitrosamines were produced in the body they would not necessarily produce tumours at the site of synthesis. Di-n-butyl nitrosamine, when injected subcutaneously, produced oesophagal, liver and urinary bladder tumours in the rat and the nitrosamine was found as the hydroxylated glucuronide in the urine (Druckrey *et al.*, 1967).

The significance of nitrosamines as human carcinogens has not yet been proved, and there are no data available on the effect of small doses of nitrosamines administered over a prolonged period in the bladder; it may be relevant that Zaldivar (1970) has shown that there is a very high incidence of cancer of the stomach, liver and oesophagus in northern Chile where there is a high level of nitrate in the drinking water and in all locally grown food.

We are indebted to the British Nutrition Foundation and the Cancer Research Campaign for financial support. We gratefully acknowledge the excellent technical assistance of Miss Deirdre Blann and the co-operation of Dr. W. Parry of the Victoria Hospital, Worksop, in obtaining the urine specimens.

### REFERENCES

Asatoor, A. M., Chamberlain, M. J., Emerson, B. T., Johnson, J. R., Levi, A. J. and Milne, M. D.—(1967) *Clin. Sci.*, **33**, 111.
Asatoor, A. M. and Simenhoff, M.—(1965) *Biochim. biophys. Acta.*, **101**, 384.
Druckrey, H., Preussmann, R., Ivankovic, S. and Schmahl, D.—(1967) *Z. Krebsforsch.*, **69**, 103.
Eisenbrand, G., Spaczynski, K. and Preussmann, R.—(1970) *J. Chromat.*, **61**, 503.
Hawksworth, G. M.—(1970) *J. med. Microbiol.*, **3**, p. ix.
Hawksworth, G. M. and Hill, M. J.—(1971) *Biochem. J.*, **122**, 28P.
Klubes, P. and Jondorf, W. R.—(1971) *Res. Commun. chem. Path. Pharmac.*, **2**, 24.
Magee, P. N. and Barnes, J. M.—(1967) *Adv. Cancer Res.*, **10**, 163.
Mirvish, S.—(1970) *J. natn. Cancer Inst.*, **44**, 633.
Preussmann, R., Neurath, G., Wulf-Lorentzen, G., Daiber, D. and Hengy, H.—(1964) *Z. analyt. Chem.*, **202**, 187.
Sander, J.—(1968) *Hoppe-Seyler's Z. physiol. Chem.*, **349**, 429.
Sander, J., Schweinsberg, F. and Menz, H.—(1968) *Hoppe-Seyler's Z. physiol. Chem.*, **349**, 1691.
Sen, N. P., Smith, D. C. and Schwinghamer, L.—(1969) *Fd Cosmet. Toxic.*, **7**, 301.
Vasak, O.—(1966) *Analytical Abstracts*, **13**, No. 5004.
Walton, G.—(1951) *Am. J. publ. Hlth*, **41**, 986.
Zaldivar, R.—(1970) *Z. Krebsforsch.*, **75**, 1.